# Supplementary material for: Predicting visual function by interpreting a neuronal wiring diagram
Source: Nature. 2024 Oct 2;634(8032):113–23. doi: 10.1038/s41586-024-07953-5 (PMC11446822; doi:10.1038/s41586-024-07953-5)
Supplement: Supplementary file 6 — CRF and ERF predictions for individual TmY4 and TmY9 cells. Analogous to Supplementary Data 3, but for TmY target types. Shown are the top four monosynaptic pathways, the strongest pathway passing through each of the top ten intermediary types (ranking from Extended Data Fig. 7), and the trisynaptic pathway Tm1–TmY–Dm3–TmY (see the section entitled Prediction of spatial normalization). [file 41586_2024_7953_MOESM6_ESM.zip › DataS4/TmY9qΓèÑ/720575940637649806.pdf]

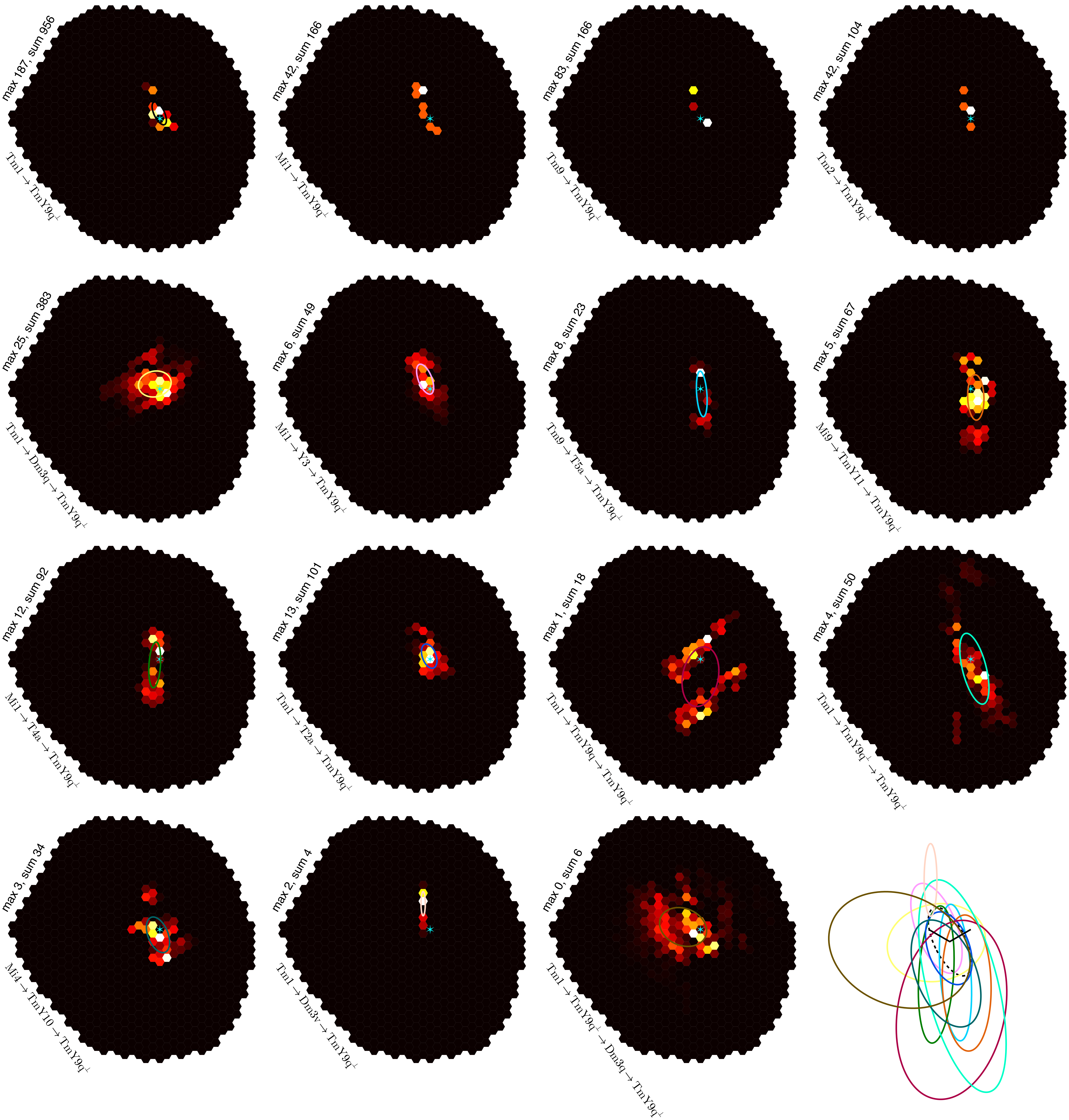
max 187, sum 956
Tm1 → TmY9q⊥
max 42, sum 166
Mi1 → TmY9q⊥
max 83, sum 166
Tm9 → TmY9q⊥
max 42, sum 104
Tm2 → TmY9q⊥
max 25, sum 383
Tm1 → Dm3q → TmY9q⊥
max 6, sum 49
Mi1 → Y3 → TmY9q⊥
max 8, sum 23
Tm9 → T5a → TmY9q⊥
max 5, sum 67
Mi9 → TmY11 → TmY9q⊥
max 12, sum 92
Mi1 → T4a → TmY9q⊥
max 13, sum 101
Tm1 → T2a → TmY9q⊥
max 1, sum 18
Tm1 → TmY9q → TmY9q⊥
max 4, sum 50
Tm1 → TmY9q⊥ → TmY9q⊥
max 3, sum 34
Mi4 → TmY10 → TmY9q⊥
max 2, sum 4
Tm1 → Dm3v → TmY9q⊥
max 0, sum 6
Tm1 → TmY9q⊥ → Dm3q → TmY9q⊥
